# Executive and semantic processes in reappraisal of negative stimuli: insights from a meta-analysis of neuroimaging studies

**DOI:** 10.3389/fpsyg.2015.00956

**Published:** 2015-07-13

**Authors:** Irene Messina, Simone Bianco, Marco Sambin, Roberto Viviani

**Affiliations:** ^1^Department of Philosophy, Sociology, Education and Applied Psychology, University of PaduaPadova, Italy; ^2^Department of Psychiatry and Psychotherapy III, University of UlmUlm, Germany; ^3^Institute of Psychology, University of InnsbruckInnsbruck, Austria

**Keywords:** emotion regulation, reappraisal, reappraisal via perspective-taking, reappraisal of stimuli, ALE, meta-analysis

## Abstract

Neuroimaging investigations have identified the neural correlates of reappraisal in executive areas. These findings have been interpreted as evidence for recruitment of controlled processes, at the expense of automatic processes when responding to emotional stimuli. However, activation of semantic areas has also been reported. The aim of the present work was to address the issue of the importance of semantic areas in emotion regulation by comparing recruitment of executive and semantic neural substrates in studies investigating different reappraisal strategies. With this aim, we reviewed neuroimaging studies on reappraisal and we classified them in two main categories: reappraisal of stimuli (RS) and reappraisal via perspective taking (RPT). We applied a coordinate-based meta-analysis to summarize the results of fMRI studies on different reappraisal strategies. Our results showed that reappraisal, when considered regardless of the specific instruction used in the studies, involved both executive and semantic areas of the brain. When considering different reappraisal strategies separately, in contrast, we found areas associated with executive function to be prominently recruited by RS, even if also semantic areas were activated. Instead, in RPT the most important clusters of brain activity were found in parietal and temporal semantic areas, without significant clusters in executive areas. These results indicate that modulation of activity in semantic areas may constitute an important aspect of emotion regulation in reappraisal, suggesting that semantic processes may be more important to understand the mechanism of emotion regulation than previously thought.

## Introduction

Emotion regulation plays a key role for the capacity, unique to humans, to react in a flexible way to emotional events (Thayer and Lane, 2000, pp. 201–216; Rozanski and Kubzansky, 2005, pp. S47–S53). Indeed, the capacity to adaptively regulate negative emotion seems to be a protective factor against the development and maintenance of psychopathology (Aldao and Nolen-Hoeksema, 2010, pp. 974–983; Aldao et al., 2010, pp. 217–237; Berking and Wupperman, 2012, pp. 128–134). Moreover, the improvement of adaptive emotion regulation skills is one of the most important aims in several psychotherapeutic treatment approaches (Greenberg and Pascual-Leone, 2006, pp. 611–630; Berking et al., 2008, pp. 1230–1237) and interventions in health psychology (Cameron and Jago, 2008, pp. 215–221; Smyth and Arigo, 2009, pp. 205–210). Especially in neuroscience, the concept of emotion regulation is key to explaining brain functioning alterations associated to psychopathology (Taylor and Liberzon, 2007, pp. 413–418) and their normalization due to psychotherapy (DeRubeis et al., 2008, pp. 788–796; Messina et al., 2013, p. e74657).

Individuals may use different emotion regulation skills to change the spontaneous flow of emotional reactions. For example, following stressful events individuals may attempt to positively reappraise the event (Weber et al., 2014, pp. 345–360), use humor (Harm et al., 2014, pp. 1895–1909), avoid thoughts associated to such events by suppressing them (Wenzlaff and Wegner, 2000, pp. 59–91), or may ruminate on the stressful event (Watkins, 2008, p. 163). Differences between emotion regulation strategies are therefore of great interest in the context of models of mental health and psychological intervention.

Among emotion regulation strategies, reappraisal has been defined as “*construing a potentially emotion-eliciting situation in non-emotional terms*” (Gross, 2002, pp. 281–291). The importance of reappraisal is due to its adaptive value in decreasing emotional experiences in response to negative events. For example, the habitual use of reappraisal has been found to be associated with the increased expression of positive emotion, more effective interpersonal functioning and increased well-being (Gross, 2002, pp. 281–291; Gross and John, 2003, pp. 348–362). In developmental psychology, evidence from longitudinal studies suggests that the habitual use of reappraisal predicts the development of interpersonal flexibility, interpersonal openness and stronger social connections in children and adolescents (English et al., 2012, pp. 780–784; Xia et al., 2014, pp. 779–786). In clinical psychology, it has been shown that reappraisal is positively correlated with mental health and negatively with emotional disorders (Aldao and Nolen-Hoeksema, 2010, pp. 974–983; Hu et al., 2014, pp. 341–362).

Several recent studies have used neuroimaging to clarify the neural mechanisms mediating reappraisal. In most of these studies, participants were exposed to negative emotional stimuli and were instructed to use emotion regulation strategies to regulate their emotional response to them (Ochsner et al., 2002, pp. 1215–1229). Using this methodology, neural correlates of emotion regulation have been identified as increases of activation in several prefrontal areas, such as the dorsolateral prefrontal cortex (dlPFC), the dorsal anterior cingulate (dACC) and the ventromedial prefrontal cortex (vmPFC) (Ochsner and Gross, 2008, pp. 153–158; Buhle et al., 2014, pp. 2981–2990). Accompanying these increases, the decrease of amygdala activation has been reported as a correlate of successful regulation of negative emotions (Diekhof et al., 2011, pp. 275–285). The involvement of prefrontal areas has been consistently reported in tasks that recruit executive attention and working memory, the control mechanisms that supervise the activation of various cognitive sub-processes through voluntary attention (Kane and Engle, 2002, pp. 637–671; Wager and Smith, 2003, pp. 255–274; Owen et al., 2005, pp. 46–59). Building on these well-established models of executive function, neurobiological models of reappraisal have focused on the concept of cognitive control to characterize the key process involved in reappraisal when responding to emotional stimuli (Ochsner and Gross, 2005, pp. 242–249; Ochsner et al., 2005, pp. 797–814). In this form of reappraisal, voluntary attention is directed to modulate responses to perceived emotional stimuli, instead of letting automatic reactions alone determine behavioral, physiological and experiential responses.

One outstanding question concerns the involvement of semantic areas in emotion regulation. Semantic processes include attributes of cognitive representations that are based on the generalization of experiences in the interaction with the environment and are subsequently used to give meaning to the new experience (Tulving, 1972, p. 4). In emotion regulation studies, temporal, inferior parietal and ventromedial prefrontal areas have been reported as activated during reappraisal tasks (Buhle et al., 2014, pp. 2981–2990). These areas are considered part of the semantic system (Patterson et al., 2007, pp. 976–987; Binder et al., 2009, pp. 2767–2796), which is the neural substrate of mental functions that allow the formation and recovery of conceptual knowledge, including representations of elements that are relevant in the context of emotion regulation such as generalization of emotional experiences (Neumann and Lozo, 2012, p. 223) and relationships that govern social interactions (Gobbini et al., 2004, pp. 1628–1635; Zahn et al., 2007, pp. 6430–6435).

The present study extends previous meta-analyses of reappraisal studies (Diekhof et al., 2011, pp. 275–285; Buhle et al., 2014, pp. 2981–2990; Kohn et al., 2014, pp. 345–355) by addressing the issue of the involvement of executive and semantic systems in emotion regulation of negative stimuli and by comparing recruitment of executive and semantic neural substrates in studies investigating different reappraisal strategies. We separately considered two different strategies of reappraisal [reappraisal of stimuli (RS) and reappraisal via perspective-taking] and performed a contrast analysis to examine the existence of significant statistical differences between the two sets of studies. The importance of this analysis stems from the consequent refinement of current neurobiological models of reappraisal, which focus on the concept of cognitive control. In contrast, the present study suggests that brain systems associated with cognitive control are not central in all forms of reappraisal, and at least another neural network – the semantic system – should be considered in emotion regulation. With this aim, we analyzed separately studies of different reappraisal strategies and we performed a contrast analysis to statistically compare these different strategies.

Our attempt to compare different emotion regulation strategies required addressing a taxonomic issue that had remained unresolved despite the increasing number of neuroimaging studies on emotion regulation. Neuroimaging studies on reappraisal provide similar instructions to investigate different strategies and different instructions to investigate similar reappraisal strategies. For example, two studies that both aimed to evaluate the effect of reappraisal of emotional stimuli provided participants with quite different instructions. Eippert et al. (2007, pp. 409–423) asked participants to “*decrease their emotional reactions by distancing themselves from the picture, by becoming a detached observer through thinking that the depicted situation is not real, only a picture*” (p. 412), but specified that “*subjects were told not to substitute negative emotions with positive emotion*” (p. 412). In contrast, Phan et al. (2005, pp. 210–219) asked participants to “*transform the scenario depicted into positive terms (e.g., women crying outside of a church could be alternatively interpreted as expressing tears of joy from wedding ceremony rather than of sorrow from a funeral)*” (p. 211). However, similar instructions have been used to evaluate different strategies of emotion regulation. For example, to investigate the strategy used in suppression, Lévesque et al. (2003, pp. 502–510) have used a typical instruction used in reappraisal studies: “*to suppress any emotional reaction to the sad stimuli. That is, they had to voluntarily decrease the intensity of the sad feelings felt in response to the sad film excerpts. To accomplish that goal, subjects were encouraged to distance themselves from those stimuli (i.e., to become a detached observer)*” (p. 503).

The need for taxonomy of reappraisal strategies was addressed in a meta-analysis of behavioral studies on emotion regulation conducted by Webb et al. (2012, p. 775). In this work three different kind of reappraisal were described: (1) RS, in which, participants are instructed to reappraise the situation or the cause of the stimulus; (2) reappraisal via perspective taking (RPT), in which participants are instructed to take another perspective (usually the perspective of a detached observer); (3) reappraisal of emotion, in which participants are instructed to interpret the emotion associated to experimental stimuli by accepting their emotional experience. Using this classification, Webb et al. (2012) were able to detect differences between specific reappraisal strategies. For example, reappraising using perspective taking proved to be more effective than reappraising the emotional stimulus or the emotional response in influencing emotional experience and expression. However, with the exception of this study, the comparison between different reappraisal strategies has been neglected in both behavioral and neuroimaging investigations.

In the present work we used the classification proposed by Webb et al. (2012, p. 775) to systematically review neuroimaging studies on reappraisal and shed light on the specific aspects of different strategies of reappraisal by investigating possible differences in the associated neural substrates. We adopted a coordinate-based meta-analytic technique specifically developed for neuroimaging studies, the Activation Likelihood Estimation (ALE) method (Laird et al., 2005, pp. 155-164), to quantify the results of fMRI studies on different reappraisal strategies.

## Materials and Methods

### Studies Selection and Classification

Neuroimaging studies on reappraisal were collected through searches in PUBMED^1^ and Google Scholar^2^ using the keywords “emotion regulation neuroimaging” or “affective regulation neuroimaging.” Additional studies were obtained reviewing the references of papers founded on PUBMED database.

We included all the papers in accordance with the following criteria: (a) use of fMRI to investigate neural correlates of reappraisal; (b) use of general linear models to analyze contrasts between reappraisal conditions versus control condition and viceversa; (d) involvement of adult healthy participants; (e) activation foci reported in 3D coordinates (x, y, z) in stereotactic space. Despite the presence of studies that have focused their analyses on specific Regions of Interest (ROIs) and may therefore bias the detection of cerebral areas (Ragland et al., 2009), we included both whole brain and ROIs analyses because of the exiguous number of studies that have investigated the whole brain activity in reappraisal (see **Table 1**). Exclusion criteria were: (a) studies investigating emotion regulation strategies different than reappraisal; (b) studies investigating reappraisal of positive emotional stimuli; (c) studies investigating reappraisal with the purpose of increasing emotional responses. Following these criteria a total of 21 studies and 437 participants were found (the main features of selected studies are shown in **Table 1**).

**Table 1:** Main features of studies included in the meta-analysis.

Studies	*N*	Emotion	Stimuli	Experimental Task Instruction	Control Task Instruction	Strategy	Whole brain/ROIs^∗^	N Foci
Banks et al. (2007)	14	Negative	Pictures	“To reinterpret the content of the picture so that it no longer elicited a negative response” (p. 305)	“During the Maintain ask, participants were instructed to attend to, be aware of and experience naturally (without trying to change or alter) the emotional state elicited by the pictures; they were told to maintain the evoked affect for the entire task block” (pp. 304–305)	*nc*	WB	9
Domes et al. (2010)	33	Negative	Pictures	“To image that the situation was not real or that they were a detached observer” (p. 760)	“Maintain trials required attentive viewing of the pictures without trying to alter the affective reaction” (p. 760)	*nc*	WB	17
Eippert et al. (2007)	24	Fear	Pictures	“Becoming a detached observer through thinking that the depicted situation is not real, only a picture” (p. 412)	“Subjects should view the picture attentively without trying to alter their emotional reactions” (p.412)	*nc*	ROIs	11
Erk et al. (2010)	17	Negative	Pictures	“To look at the following picture directly but try to take the position of a detached observer, thinking about the present picture in a neutral way” (p. 15727)	“Look at the following picture directly and permit feeling your emotions” (p. 15727)	RPT	WB/ ROIs	11
Goldin et al. (2008)	17	Disgust	Videos	“Thinking objectively to decrease emotional reactivity to films, for example, by assuming the perspective of a medical professional watching an instructional video or focusing on technical aspects of the film” (p. 578)	No instruction	RPT	WB	35
Hayes et al. (2010)	25	Negative	Pictures	“Place yourself as an observer in the scene, but change the way you think about it by making it not relevant to you or your loved ones” (p. 3)	“Simply look at the picture and let any emotions you’re feeling unfold naturally” (p. 3)	RS	ROIs	23
Kanske et al. (2011)	25	Negative	Pictures	“Decrease any emotional response by reinterpreting the displayed situation, for example, as produced by actors and therefore not real, as meaning something else, or having a different outcome than initially suggested by the picture” (Kanske et al., 2011, p. 1380)	“Participants attended the content of the picture but did not manipulate the emotional response to it” (Kanske et al., 2011, p. 1380)	RS	ROIs	24
Kim and Hamann (2007)	10	Negative	Pictures	“Imagining the scenes as less personally relevant (e.g., dissociating themselves from the main figures), imagining the scenes as unreal, and imagining the scenes as physically farther away from themselves” (pp. 777-778)	“participants were instructed to view the picture in a natural way and not to try to change the emotion elicited by the picture” (p. 777)	RS	WB/ ROIs	40
Koenigsberg et al. (2010)	16	Negative	Pictures	“Relate to the image as though they were not personally connected in any way to the pictured individuals or the context in which they were situated, i.e., as though they were an anthropologist viewing the scene objectively or an emergency room doctor maintaining a detached clinical perspective so that he can function coolly in the situation” (p. 1815)	“(Subject) were to simply allow themselves to experience whatever emotion the picture spontaneously evoked in them” (p. 1815)	RPT	WB	11
Kross et al. (2009)	16	Negative	Sentences	“to recognize that the feelings they experienced during recollection were passing mental events that were psychologically distant from the self and did not control them” (p. 361)	“The first “feel” strategy directed individuals to focus on the specific feelings that naturally flowed through their mind as they thought about their recalled experiences” (p. 361)	*nc*	WB	6
Lévesque et al. (2003)	20	Sadness	Videos	“Voluntarily decrease the intensity of the sad feelings felt in response to the sad film excerpts. To accomplish that goal, subjects were encouraged to distance themselves from those stimuli (i.e., to become a detached observer)” (p. 503)	“Subjects were instructed to react normally to the sad film excerpts, that is, to allow themselves to become sad in response to these stimuli” (p. 503).	RPT	WB/ ROIs	11
McRae et al. (2008)	25	Negative	Pictures	“Reinterpretations were limited to three categories: (1) It’s not real (e.g., it’s just a scene from a movie, they’re just pretending), (2) Things will improve with time (e.g., whatever is going wrong will resolve over time), (3) Things aren’t as bad as they appear to me (e.g., the situation looks worse than it is, it could be a lot worse, at least it’s not me in that situation)” (p. 148)	No instruction	RS	WB/ROIs	16
McRae et al. (2010)	18	Negative	Pictures	“To reinterpret the situation depicted in the picture in a way that made them feel less negative about it. When reappraising, participants used the instructed strategy of reinterpreting the affects/dispositions, outcomes, and contexts depicted in images” (p. 249)	“Participants were instructed to pay attention and respond naturally to the subsequent stimulus, allowing themselves to have whatever reaction the picture would normally evoke in them” (p. 249)	*nc*	WB/ROIs	19
Modinos et al. (2010)	18	Negative	Pictures	“To reinterpret its content so that it no longer elicited a negative response” (p. 371)	“Subjects were instructed to attend and naturally experience any feelings elicited by the photo” (p. 371)	*nc*	WB/ROIs	10
Ochsner et al. (2002)	15	Negative	Pictures	“Participants were instructed to reinterpret the photo so that it no longer elicited a negative response. Reappraisal was commonly accomplished by generating an interpretation of, or a story about, each photo that would explain apparently negative events in a less negative way (e.g., women depicted crying outside of a church could be described as attending a wedding instead of a funeral)” (p. 1225)	“Participants were instructed to attend to and be aware of, but not to try to alter, any feelings elicited by it” (p. 1225)	RS	WB/ROIs	19
Ochsner et al. (2004)	24	Negative	Pictures	“Participants in the self-focus group were instructed to increase their sense of objective distance, viewing pictured events from a detached, third-person perspective. To decrease negative emotion participants could view the sick person from the detached; clinical perspective of one not personally connected in any way to the pictured individual and the context in which she is situated. Participants assigned to the situation-focused group were instructed to reinterpret the emotions, actions, and outcomes of individuals as depicted in their situational context. To decrease negative emotion, participants in the situation-focused group were asked to imagine pictured events getting better” (pp. 484–485)	“Participants were instructed simply to look at the image and let they respond naturally” (p. 485)	RPT	ROIs	40
Phan et al. (2005)	14	Negative	Pictures	“To decrease voluntarily the intensity of their negative affect by using the cognitive strategy of reappraisal which is to reinterpret the content of the picture so that it no longer elicited a negative response […] Two main examples of cognitive reappraisal were provided to facilitate understanding of the strategy: (1) transforming the scenario depicted into positive terms (e.g., women crying outside of a church could be alternatively interpreted as expressing tears of joy from wedding ceremony rather than of sorrow from a funeral) and (2) rationalizing or objectifying the content of the pictures (e.g., a woman with facial bruises could be translated as an actor wearing makeup rather than a victim of domestic abuse)” (p. 211)	“Subjects were instructed to attend to, be aware of, and experience naturally (without trying to change or alter) the emotional state elicited by the pictures; they were told to maintain the evoked affect for the entire block” (p. 211)	RS	ROIs	18
Schardt et al. (2010)	37	Fear Disgust	Pictures	“To look at the picture while detaching yourself from any emotional response which may arise by adopting the position of a detached observer, who is not affected by the scene presented in the picture” (p. 945)	“Participants were instructed to look at the picture and permit you to feel whichever emotional response arises naturally, without trying to alter it” (p. 945)	RPT	WB, ROIs	22
Urry et al. (2006)	19	Negative	Pictures	“To reduce the intensity of their negative affect, for which they were trained to either (1) view the situation as fake or unreal, or (2) imagine that the situation being depicted had a different outcome than the one suggested (e.g., victims of a car accident survived and healed well)” (p. 4416)	“Participants were instructed to maintain their attention to the picture without changing their negative affective experience” (p. 4416)	RS	WB/ROIs	2
Wager et al. (2008)	30	Negative	Pictures	“Participants viewed aversive images, and were asked to reappraise the emotional value of those images so that the emotional impact was less negative. More specifically, they were instructed to generate a positive interpretation of the scene depicted in each picture that reduced the emotional impact” (p. 1048)	“Participants were asked to view the image, understand its content, and allow they to experience/feel any emotional response it might elicit” (p. 1048)	RS	WB/ROIs	10
Walter et al. (2009)	20	Negative	Pictures	“Subjects were instructed to intentionally regulate their emotions by taking the position of a neutral observer. More specifically they were instructed to: “Look at the following picture directly but try to take the position of a Non-involved observer, thinking about the present picture in a neutral way” (p. 2)	“Subjects were instructed to simply watch the pictures and permit all upcoming emotions. More specifically they were instructed to: “Look at the following picture directly and permit feeling your emotions” (p. 2)	RPT	WB/ROIs	14

nc, not classified; RST, reappraisal via perspective-taking; RS, reappraisal of stimuli; WB, whole brain analyses; ROIs, regions of interest analysis. *In case of Whole brain/ROIs studies, all foci of brain activation have been considered in the meta-analysis.

A careful coding of emotion regulation instructions that participants received in the neuroimaging studies was followed (see **Table 1** for original instructions of experimental and control tasks reported in single studies). Thus, according to the taxonomy proposed by Webb et al. (2012, pp. 775), we classified reappraisal strategies as: (1) Reappraisal of stimulus in which participants are instructed to reappraise the situation or the cause of the stimulus, for example thinking that it is not real (RS; *N* = 8); (2) RPT in which participants are instructed to take the perspective of a detached observer; (RPT; *N* = 7); (3) Reappraisal of emotional response (*N* = 1), in which participants are instructed to interpret the emotion associated to experimental stimuli in a mindfulness manner (Kross et al., 2009, pp. 361–366); (4) Reappraisal not-specified (*N* = 5), when instructions were generic or included more strategies. Instructions for control tasks were quite similar for all studies and they consisted in natural responses to experimental stimuli.

### Meta-Analytic Procedure

Several meta-analyses were carried out based on the classification described in the previous section. Firstly, a preliminary meta-analysis aimed at evaluating the neural correlates of reappraisal regardless of the specific instructions. Secondly, on the basis of the classification of the instructions, we conducted two separate analyses to verify the existence of specific neural correlates of RPT and reappraisal of the stimulus. Because we found only one study investigating reappraisal of emotion (Kross et al., 2009, pp. 361–366), we were not able to explore neural correlates of this strategy in a separate meta-analysis.

To conduct the meta-analyses, the ALE method for coordinate-based meta-analysis of neuroimaging data was used (Eickhoff et al., 2009, pp. 2907–2926). This methods is based on the evaluation of the overlap between foci of activation found in different studies and treats the reported foci not as single points, but as centers for 3D Gaussian probability distributions capturing the spatial uncertainty (Turkeltaub et al., 2002, pp. 765–780; Turkeltaub et al., 2012, pp. 1–13). To this aim, an algorithm is used to identify clusters of brain activity that show a convergence of activation across experiments and determine if the clusters thus obtained occur more frequently than in the null distribution arising from random spatial association between the results of different experiments. ALE meta-analysis was carried out using GingerALE 2.3 software distributed by the BrainMap project^3^ (Laird et al., 2005, pp. 155–164). We employed the “non-additive” method, which models each focus with a Gaussian function defined by a full-width at half-maximum (FWHM) kernel size empirically determined by finding the maximum across each focus’s Gaussian (Turkeltaub et al., 2012, pp. 1–13). The non-additive method allows the modeling of the spatial uncertainty of each focus arising from inter-subject and inter-study variability. The meta-analyses were performed in Talairach space. Coordinates reported in studies in Montreal Neurological Institute (MNI) space were transformed into Talairach coordinates using the Lancaster transform, icbm2tal algorithm (Laird et al., 2010, pp. 677–683) included in the Convert Foci tool of GingerALE.

We conducted several meta-analyses. In the first meta-analysis we included all reappraisal studies irrespective of the strategy. Foci of activation were collected from all the contrasts between reappraisal conditions versus control conditions and vice versa, as reported in the original studies. In the second meta-analysis we considered the contrasts between RS *versus* the control condition and vice versa. In the third meta-analysis we considered the contrasts between reappraisal via perspective-taking *versus* the control condition and vice versa. Finally, to test the interaction between condition (experimental condition *versus* control condition) and strategy (RS *versus* reappraisal via perspective-taking), a subtractive analysis was conducted comparing ALE values of specific reappraisal strategies (RS versus RPT; Eickhoff et al., 2011, pp. 938–949). In all cases statistical significance was determined through a permutation tests.

## Results

### Neural Correlates of Reappraisal

**FIGURE 1:**
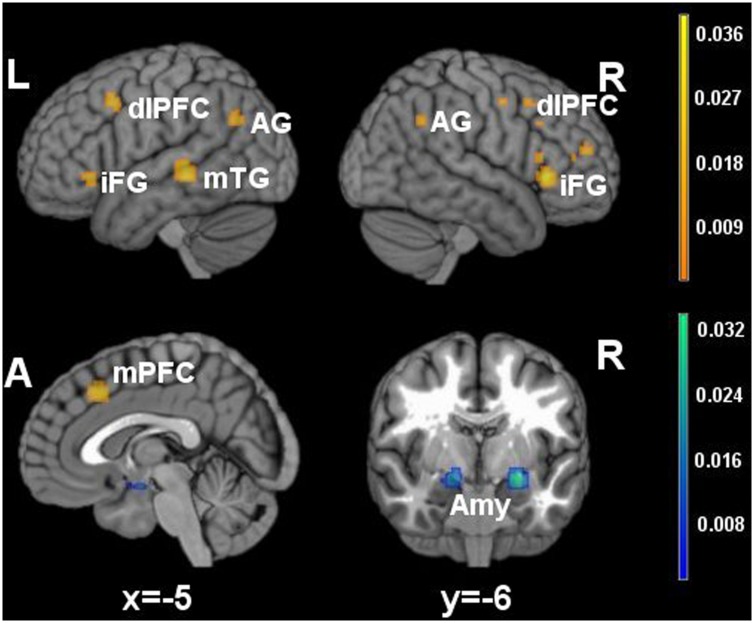
**Brain activity in reappraisal tasks.** In warm colors significant cluster of increased brain activity, in cold colors significant clusters of decrease brain activity. mPFC, medial prefrontal cortex; dlPFC, dorsolateral prefrontal cortex; iFG, inferior frontal gyrus; mTG, middle temporal gyrus; AG, angular gyrus; Amy, amygdala.

The first meta-analysis evaluated the main effect of reappraisal regardless of the specific reappraisal instruction used in the studies (see **Table 2**, **Figure 1**). This analysis was based on 21 studies and 437 participants, yielding a total of 245 foci for the contrast reappraisal condition versus control condition and 13 studies, 272 participants, yielding a total of 73 foci for the contrast control condition versus reappraisal condition. The probability maps were thresholded at *p* < 0.001 and corrected using false discovery rates (FDRs), the minimum clusters extent was of 200 mm × 200 mm × 200 mm.

**Table 2:** Significant clusters of brain activity in reappraisal studies.

Cluster	Areas	Talairach Coordinates			Brodmann’s areas	Cluster size (mm^3^)	ALE score
		*x*	*y*	*z*
**(A) Reappraisal versus Control Task (*p* < 0.001, FDR correction)**		
(1)	Medial prefrontal cortex/dorsal anterior cingulate	4	22	42	32/8/6	1008	0.031
(2)	Middle temporal gyrus/superior temporal gyrus	-58	-34	-2	21/22/42	864	0.029
(3)	Inferior frontal gyrus/middle frontal gyrus	46	26	0	47/45/13	840	0.037
(4)	Dorsolateral prefrontal cortex	-38	12	44	6/8/9	400	0.023
(5)	Inferior frontal gyrus	-46	26	-6	47/45	320	0.026
(6)	Angular gyrus/middle temporal gyrus/inferior parietal lobe	-46	-66	32	39	296	0.022
**(B) Control Task versus Reappraisal (*p* < 0.001, FDR correction)**							
(1)	Amygdala/putamen	-26	-2	-14	34	1288	0.033
(2)	Parahippocampal gyrus/amygdala	12	-12	-16	34	704	0.024

*ALE, activation likelihood estimation; FDR, false discovery rate.*

Significant clusters of increased activation were found in the dorsal attentional system (dlPFC and the posterior medial prefrontal cortex, with extension to the anterior cingulated cortex). Several clusters of increased activation were located also in the semantic system, i.e., in the inferior prefrontal gyrus, superior/middle temporal gyrus and in the angular gyrus on the left side. Finally, clusters of decreased activation were found in limbic areas such as the amygdala and the parahippocampal gyrus.

### Neural Correlates of Specific Reappraisal Strategies

Meta-analyses on the effect of specific strategies were conducted considering RS and RPT separately. For both meta-analyses, probability maps were thresholded at *p* < 0.01 and corrected using FDRs.

**FIGURE 2:**
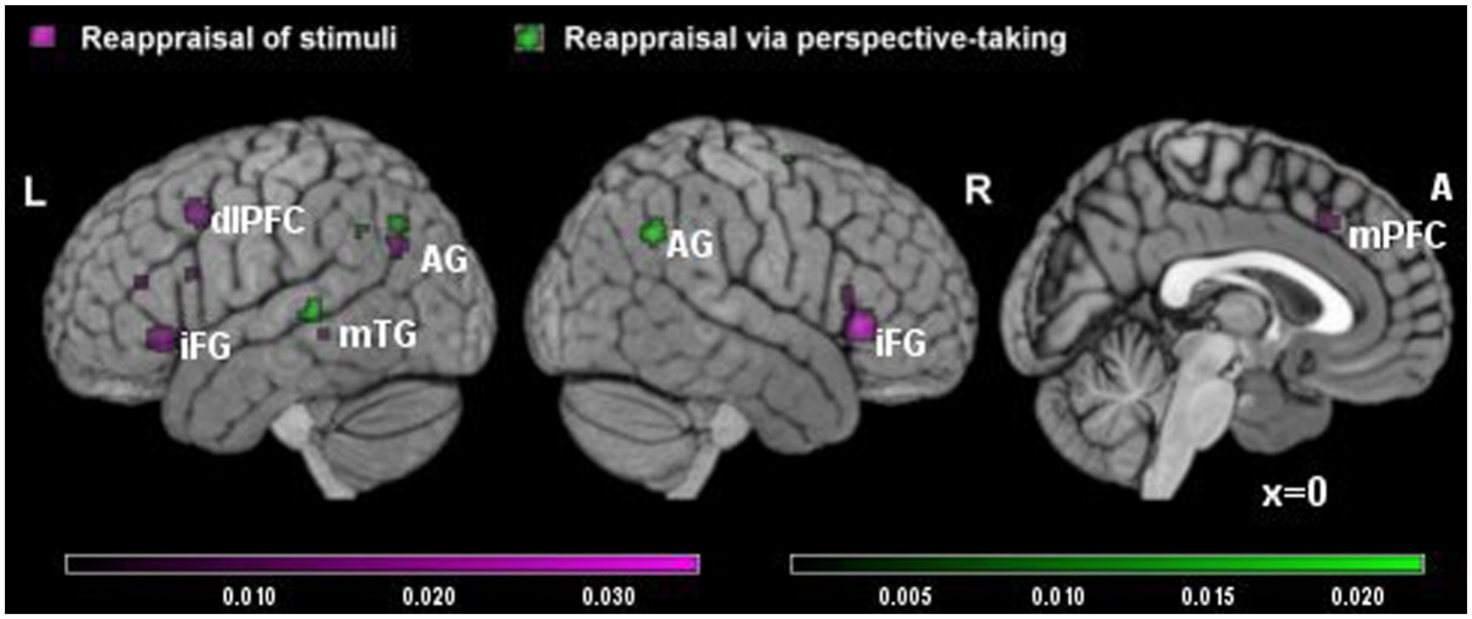
**Brain activity in reappraisal of stimuli and reappraisal via perspective-taking.** In violet increased brain activity during RS; in green increased brain activity during reappraisal via perspective-taking. mPFC, medial prefrontal cortex; dlPFC, dorsolateral prefrontal cortex; iFG, inferior frontal gyrus; mTG, middle temporal gyrus; AG, angular gyrus.

The meta-analysis of studies on RS included eight studies and 163 participants, yielding a total of 105 foci for the contrast RS condition versus control condition and four studies, 73 subjects yielding a total of 18 foci for the contrast control condition versus RS. The results of this meta-analysis were quite similar to the results of the main meta-analysis, with significant clusters of increased brain activation in dorsal attentional system (in dorsolateral and medial prefrontal cortex) and in the sematic system (temporal gyrus and angular gyrus on the left, inferior prefrontal cortex; see **Table 3A**, **Figure 2** in violet), and significant clusters of decreased activation in areas involved in emotional reactivity (amygdala and parahippocampal gyrus bilaterally; see **Table 3B**, **Figure 2** in violet).

**Table 3:** Significant clusters of brain activity in RS and (A,B) and reappraisal via perspective-taking (RPT; C,D).

Cluster	Areas	Talairach coordinates			Brodmann’s areas	Cluster size (mm^3^)	ALE score
		*x*	*y*	*z*			
**(A) Reappraisal of stimuli versus control task (*p* < 0.01, FDR correction)**			
(1)	Inferior frontal gyrus/middle frontal gyrus	44	26	0	13/47/45	1232	0.034
(2)	Medial prefrontal cortex/dorsal anterior cingulate	6	20	44	6/8	744	0.016
(3)	Dorsolateral prefrontal cortex	-40	12	44	6/8/9	704	0.018
(4)	Inferior frontal gyrus	-46	26	-6	47/45	544	0.021
(5)	Medial prefrontal cortex/dorsal anterior cingulate	-8	14	46	32/6/8	400	0.016
(6)	Middle temporal gyrus	-56	-38	-4	20/22	304	0.014
(7)	Angular gyrus/middle temporal gyrus	-46	-66	32	39	264	0.015
**(B) Control task versus reappraisal of stimuli (*p* < 0.01, FDR correction)**			
(1)	Parahippocampal gyrus/amygdala	-22	-8	-12	34	472	0.016
(2)	Parahippocampal gyrus/amygdala	16	-8	-12	34/28	360	0.011
**(C) Reappraisal via perspective-taking versus control task (*p* < 0.01, FDR correction)**			
(1)	Inferior parietal lobule/angular gyrus	50	-56	36	39/40	696	0.018
(2)	Superior temporal gyrus/ middle temporal gyrus	-58	-34	6	22/42	320	0.016
**(D) Control task versus reappraisal via perspective-taking (*p* < 0.01, FDR correction)**			
(1)	Parahippocampal gyrus/amygdala/putamen	-26	-2	-14	34	848	0.021
(2)	Parahippocampal gyrus/amygdala	16	-6	-12	34/28	456	0.021
(3)	Parahippocampal gyrus/thalamus/hippocampus	-22	-28	-4	27/28	352	0.016

*ALE, activation likelihood estimation; FDR, false discovery rate.*

The meta-analysis of RPT studies included seven studies and 151 participants, yielding a total of 77 foci for the contrast RPT condition versus control condition and 51 foci for the contrast control condition versus RPT. Here, the biggest clusters of increased activation were located in the right inferior parietal lobe/angular gyrus on the right and in the superior temporal gyrus on the left (see **Table 3C**, **Figure 2** in green). No significant cluster of activation was found in the prefrontal cortex. Significant clusters of decreased activation were found in the amygdala bilaterally and thalamus (see **Table 3D**).

Finally, a comparative analysis was conducted to quantify the differences between the RS and RPT reappraisal strategies. Also in this case, probability maps were threshold at *p* < 0.01 and corrected using FDRs. Due to the small number of studies, only a cluster survived to the direct comparison between strategies. This cluster was specific for RS strategies but not for RPT. It was located in the medial prefrontal cortex (localization of the cluster in 3D Talairach coordinates: *x* = -14, *y* = 11, *z* = 54; Broadmann’s area = 6; *z*-score obtained in the subtractive analysis: *z* = 3.01, *p* < 0.01 corrected for FDR).

## Discussion

Neurobiological models of reappraisal emphasize the role of executive function in emotional control (Ochsner and Gross, 2005, pp. 242–249; Ochsner et al., 2012, pp. E1–E24), whereas the contribution of semantic processes has been less addressed in the literature. In the present study we systematically reviewed, classified and meta-analyzed neuroimaging studies on different reappraisal strategies of negative stimuli. Our attempt was to investigate the role of executive and semantic functions in emotion regulation. Namely, we verified the involvement of these functions in reappraisal regardless of the specific form of reappraisal investigated in single studies, and considering two specific reappraisal tasks on the basis of the instruction provided by authors to participants in each single study.

The classification of neuroimaging studies on reappraisal was carried out through careful coding of emotion regulation instructions that participants received in each neuroimaging study that corresponded to our selection criteria. We focused on two main categories of studies on reappraisal that resulted to be well-represented in the literature: RS and RPT. In studies on RS participants were instructed to reappraise the situation or the cause of experimental stimulus. In this case typical experimental paradigms were based on the exposure of participants to emotional negative pictures during the fMRI scanning, and they were asking to think that the picture was not real (for example, to think that the pictures showed was a movie or that the persons in the pictures were actors). In studies of reappraisal via perspective-taking participants were instructed to take the perspective of a detached observer during the exposure to negative emotional pictures.

In the main meta-analysis of reappraisal, which considered all studies regardless of the specific instruction adopted in the study, the neural substrates associated with recruitment of executive processes resulted activated by reappraisal. Significant clusters of increased activation were detected in dorsolateral and dorsomedial prefrontal/anterior cingulated areas, which are part of the voluntary attentional system (Duncan and Owen, 2000, pp. 475–483; Hopfinger et al., 2000, pp. 284–291). Accompanying such activations, clusters of decreased activity were detected in subcortical areas associated to emotional reactivity such as the amygdala (Phan et al., 2002, pp. 331–348; Sergerie et al., 2008, pp. 811–830). As in previous meta-analyses (Diekhof et al., 2011, pp. 275–285; Buhle et al., 2014, pp. 2981–2990; Kohn et al., 2014, pp. 345–355), the observed increased activation in areas of the voluntary attentional system is consistent with the neurobiological model of reappraisal, which views it as a controlled process involving executive functions and working memory (Ochsner and Gross, 2005, pp. 242–249). Furthermore, the decreased activation of limbic areas may be interpreted as diminished arousal following regulation (Banks et al., 2007, pp. 303–312; Wager et al., 2008, pp. 1037–1050). Together with these results regarding the voluntary attentional system, the meta-analyses also detected activations of areas that are considered part of the semantic system, such as the temporal lobe, inferior frontal gyrus and angular gyrus (Patterson et al., 2007, pp. 976–987; Binder et al., 2009, pp. 2767–2796). Despite also these results have been observed in previous meta-analyses (Diekhof et al., 2011, pp. 275–285; Buhle et al., 2014, pp. 2981–2990; Kohn et al., 2014, pp. 345–355), the importance of their contribution to emotion regulation has been neglected in neurobiological models of reappraisal.

In the present study, the separate consideration of RS and RPT strategies allowed us to observe the different prominence of executive and semantic areas in the reappraisal strategies. We observed that areas of the voluntary attentional system were activated in RS strategy but not in RPT. Specifically, in the analysis in which these two strategies were directly compared a significant cluster of increased activation located in the dorsomedial/anterior cingulated cortex differentiated activations in RS from RPT reappraisal. These results suggest that the RPT strategy may rely less on executive control than RS, suggesting that executive functions might be not as essential for emotion regulation as previously thought (Ochsner and Gross, 2008, pp. 153–158; Ochsner et al., 2012, pp. E1–E24).

Several considerations support this conclusion. According to the models of reappraisal as a form of cognitive control (Ochsner and Gross, 2005, pp. 242–249; DeRubeis et al., 2008, pp. 788–796), the involvement of voluntary attention in RS should be associated with increased effectiveness in emotion regulation. However, this association was not observed in the meta-analysis, where the different involvement of prefrontal areas in RS and RPT did not correspond to different outcomes in terms of limbic activation (both strategies were associated to similar decrease in amygdala activity). These results are not surprising in the light of behavioral data to the effect that both RS and RPT are similar in reducing emotional response to unpleasant stimuli (Deveney and Pizzagalli, 2008, pp. 435–444; Webb et al., 2012, p. 775). In one study RPT was even more effective in this regard, whereas RS was more likely to maintain subjective experience and facial expression associated to the emotion elicited by the experimental stimuli (Shiota and Levenson, 2012, p. 416). Due to the absence of evidence on differences in effectiveness of the reappraisal strategies considered in the present study, the variable involvement of executive functions in RS compared to RPT may simply reflect a difference in the amount of cognitive effort required by each strategy. Neuroimaging studies of working memory have reported progressive activation of areas of the voluntary attentional system in association with the cognitive demands required by the task (Rypma et al., 2002, pp. 721–731). Reappraisal may involve working memory when a cognitive effort is required, but this effort may be reduced when the strategies adopted to regulate are less demanding (Jansma et al., 2001, pp. 730–743). Furthermore, other studies report the existence of implicit forms of emotion regulation, in which emotion are regulated without voluntary attempts to control them (Mauss et al., 2007, pp. 1–18; Koole and Rothermund, 2011, pp. 389–399). The few existing neuroimaging studies on implicit forms of emotion regulation have shown that the areas of the voluntary attentional system are not recruited (Viviani et al., 2010, p. e15454) and do not correlate with individual differences in spontaneous avoidance (Benelli et al., 2012, p. 239).

In contrast to the inconstant recruitment of the voluntary attentional network, the activation of semantic areas emerged as a common aspect of emotion regulation despite of the specific strategy adopted by participants. How do semantic processes contribute to emotion regulation? The presence of specific semantic content may have a role in the effectiveness of reappraisal. It is conceivable that the existence of a wealth of semantic representations that one can activate in order to reappraise emotional stimuli facilitates the generation of alternative representations of what happened. Instead, a person with poor semantic representations of contingencies related to emotionally arousing situations may have more difficulties in using semantic information to reappraise emotional stimuli. For example, black/white thinking in borderline patients may be related to their difficulties in generating mentalizing appraisals of other people’s possible motives, a form of knowledge that is based on semantic memory for social interactions (Irish et al., 2014, pp. 1241–1253). Due to the evidence that semantic activation may play a role in emotion regulation regardless to the involvement of executive functions, our hypothesis is that semantic processes may involve different kind of attentional processes. In studies on spatial attention (Corbetta and Shulman, 2002, pp. 201–215; Chica et al., 2013, pp. 107–123), a ventral attentional network has been described that is activated when attention is directed spontaneously to stimuli that are behaviourally relevant, regardless the voluntary attempt of participants to direct their attention to this stimuli and regardless to the salience of this stimuli, but is also recruited in studies in which top–down control of emotional stimuli is required (Corbetta et al., 2008, pp. 306–324). Such network includes the temporo-parietal junction observed in RS and RPT studies meta-analyzed in the present study.

In the context of emotion regulation, the ventral network may influence emotion regulation by conveying the influence of semantic networks due to the intervention of a proactive mechanism of control on the emotional representations (Viviani, 2013). Because emotion regulation strategies such as spontaneous avoidance (Viviani et al., 2010, p. e15454; Benelli et al., 2012, p. 239) or acceptance (Kross et al., 2009, p. 361–366) also appear to recruit these areas, there is room in future studies for investigating forms of emotion regulation not based on cognitive control to enrich emotion regulation models and to clarify the adaptive value of emotion regulation strategies.

### Limitations

Several limitations of the present study should be noted. First, the majority of the studies which have investigated emotion regulation have used ROIs approach. The *a priori* definitions of ROIs may bias the detection of cerebral areas in emotion regulation literature, as the effects in ROIs that were defined *a priori* are likely to be overrated (Diekhof et al., 2011, pp. 275–285). Secondly, many of the studies investigating reappraisal used quite generic instructions that did not allow us to classify the specific strategies under investigation. This limitation had the consequence to reduce the number of studies included in meta-analyses of specific strategies. Third, we cannot exclude that participants were using the strategy of distracting themselves from the experimental stimuli (despite the instruction provided by experimenters). For example, if self-distraction involved the generation of alternative verbal material, then it would be expected to be associated with the activation of semantic areas, which has been detected in words generation tasks (Petersen et al., 1989, p. 153–170). However, as we noted in Section “Discussion,” the ready availability of appropriate alternative semantic content may be an important factor in the effectiveness of reappraisal also when following the strategy of “thinking of something else.” Hence, an important message of the present paper is that semantic activation may play a role in emotion regulation regardless of the involvement of executive functions. Future studies may define more carefully the specific reappraisal strategy under investigation. Because of the small number of studies included in meta-analyses of specific strategies, their results should be considered as explorative, but not conclusive.

## Conclusion

Both executive and semantic aspects of emotion regulation were found to be involved in functional imaging studies of emotional reappraisal, but with a different pattern according to the reappraisal strategy. In reappraisal of emotional stimuli, executive functions were found to be recruited in the meta-analysis, even if also semantic areas were activated. Instead, in RPT the most important clusters of brain activity were found on parietal and temporal semantic areas, similarly to less adaptive strategies such as suppression or avoidance. This heterogeneity suggests that executive functions are just one aspect of emotion regulation, and that their relevance may depend on the specific reappraisal strategy adopted by participants, including the depth of semantic encoding and the use of semantic networks.

## Conflict of Interest Statement

The authors declare that the research was conducted in the absence of any commercial or financial relationships that could be construed as a potential conflict of interest.

## Footnotes

1 http://www.ncbi.nlm.nih.gov/pubmed/

2 http://scholar.google.com/databases

3 http://brainmap.org/ale/
